# A study on the assessment and scoring standard of badminton course in colleges and universities: A review

**DOI:** 10.1097/MD.0000000000032230

**Published:** 2022-12-09

**Authors:** Xiaofeng Hu, Qiaolin Jiang

**Affiliations:** a Faculty of Table tennis, badminton and tennis, School of Sports Training, Chengdu Sport University, Chengdu, P.R. China; b Chongqing No. 11 Middle School, Chongqing, P.R. China.

**Keywords:** assessment, badminton class, school physical education, scoring standard

## Abstract

With the continuous promotion of quality education, the importance of physical education in college teaching is increasingly prominent. In order to meet the sports needs of different students, badminton courses are introduced in colleges and universities. Badminton not only has a strong entertainment, but also a good physical exercise, loved by students. However, due to the existing badminton class evaluation standard is not scientific and reasonable, which is not conducive to the smooth implementation of physical education, this paper studies the current situation of badminton assessment and scoring standards, points out the existing problems in the teaching process, and gives a new way to improve the assessment and scoring standards of badminton in colleges and universities, in order to provide reference for the assessment system of badminton in colleges and universities, and then improve the teaching effect of badminton.

## 1. Introduction

Badminton is a small ball game with good fitness and entertainment. Because badminton is easy to control, it is loved by people of all ages. With the development of society, under the background of the new physical education curriculum reform, badminton has also been added to the college physical education curriculum. At the same time, the variability of badminton and the characteristics of fighting wisdom and bravery are well received by college students.^[1]^ The purpose of badminton in colleges and universities is not only to carry out an activity for students to exercise, but also for students to gain the effect of physical training. Therefore, the development of badminton course is very beneficial and it should be vigorously expanded in colleges and universities. It is necessary for teachers to teach students badminton skills through badminton teaching. With the rapid development of society, colleges and universities have higher and higher requirements for the physical quality of students, so the status of physical education in colleges and universities has gradually improved. In this context, badminton, like other traditional sports, has entered the college physical education class. As we all know, badminton is changing rapidly, and its technical requirements are more delicate than other sports, and the technical requirements for students are also strong.^[2]^ It is a great challenge for teachers to let students fully master the skills and knowledge in a very limited classroom teaching time. Therefore, teachers should make full use of badminton class time, use scientific and reasonable teaching methods, in order to better improve the classroom teaching effect. It includes not only the badminton related knowledge and skills taught by teachers, but also a good assessment and scoring system. The assessment and scoring system can reflect the teaching effect of teachers and follow up the learning effect of students in time, to provide effective feedback information for badminton teaching. Teachers can adjust the existing teaching methods, teaching progress and content distribution according to the feedback information. However, many colleges and universities in China currently use badminton assessment and evaluation methods which seriously neglect the evaluation of students in the learning process.^[3]^ Most colleges and universities use competitive assessment standards to assess students’ performance, which not only affects students’ enthusiasm for learning, but also reduces the teaching effect of Badminton.^[4]^ Therefore, it is imperative to reform the evaluation standard of badminton in Colleges and universities. It is necessary for colleges and universities to establish a good evaluation standard to improve students’ comprehensive quality through sports. At the same time, it is also the basic condition for badminton class to shine and heat up in college sports teaching. It is very necessary to promote the development of badminton course in Colleges and universities with quality education as the guiding ideology, so that badminton teaching can meet the needs of physical and mental development of contemporary college students.

In addition, developing physical education courses in Colleges and universities is to cultivate students’ comprehensive quality to better serve the society. When it comes to education and teaching, there must be assessment and scoring mechanism. The technical examination of badminton can make a corresponding measurement of the teaching effect, which is a method to test the teaching quality. Through the badminton class assessment score, students’ learning level can be assessed, and teachers’ teaching results can also be summarized. Therefore, colleges and universities for student’s badminton course assessment and scoring standards are also constantly improving. However, at present, the evaluation system of badminton adopted by most universities in China is the evaluation standard of competitive badminton. There are many shortcomings in the evaluation method, such as despise students’ interest in learning, pay attention to skills, despise process, pay attention to results, despise characteristics, pay attention to content and so on. Such assessment standards not only do not make students feel the fun of badminton, but also greatly reduce students’ interest in badminton. Therefore, according to the actual situation of students, it is very necessary for colleges and universities to establish a good assessment and scoring standard for badminton class.

## 2. The current situation of badminton assessment in colleges and universities

**Figure 1.**
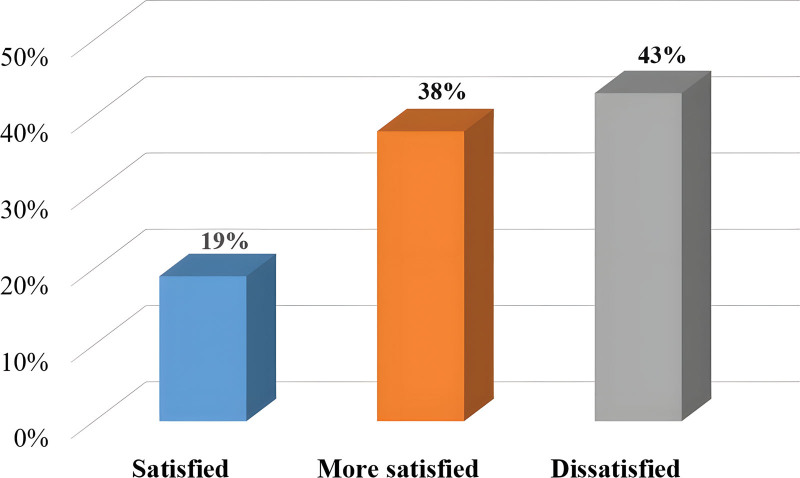
Students’ satisfaction with the current badminton assessment and scoring standards.

At present, most colleges and universities in China only need to evaluate badminton based on 3 parts: singles performance, technical evaluation, and daily performance. The singles competition is based on the ranking. Badminton technical assessment mainly includes forehand high ball and forehand hit high ball. The teacher will give technical scores according to the completion of each student. The general assessment method is that each student has 5 opportunities in each assessment item, among which 3 balls can get the full score of the item between the serve and the end line after falling into the doubles. Students’ daily performance is mainly based on attendance rate and performance in class, accounting for 20% of the total score. According to the existing literature, and the satisfaction with the badminton course questionnaire, we know that students’ satisfaction with the existing badminton course assessment and scoring standards is shown in Figure 1.

**Figure 2.**
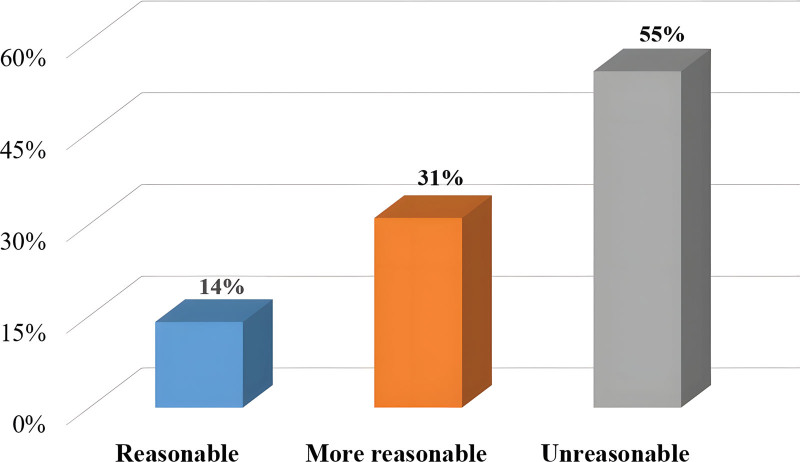
Students’ attitudes towards the assessment methods of badminton forehand sending and hitting.

According to the survey, only 19% of the students are satisfied with the current badminton assessment and scoring method, 38% of the students are satisfied with it, and 43% of the students are dissatisfied with it. The most important reason for this result is that most students think that the evaluation standard is too single. Because of the uneven physical quality of each student, it is difficult for some students with poor physical quality to achieve good results even if they work very hard. Therefore, this assessment and scoring method greatly reduces the enthusiasm of students for badminton and participation in sports. The attitude of students towards the standard method of badminton forehand sending and hitting is shown in Figure 2.

Only 14% of the students consider that the current assessment methods of badminton forehand high and far shot and hitting high and far shot are reasonable, 31% of the students hold a more reasonable attitude, and more than 50% of the students think that they are unreasonable (shown in Fig. 2). There are many problems in the current badminton assessment and scoring standards of most universities in China. Although it is an important assessment item for students to score in badminton class, most of the assessment methods are used in competitive sports center. It is very difficult for college students to pass the examination of high ball, which reduces the effectiveness of badminton course assessment.

## 3. Problems in the assessment and scoring standards of badminton in colleges and universities

### 3.1. Despise interest and value skill

Most colleges and universities in China pay too much attention to the teaching of skills. Therefore, when teachers teach badminton knowledge to students, the proportion of teaching time in terms of technology is high, seriously ignoring the cultivation of students’ physical quality. To know that students’ mastery of badminton technology needs good physical quality as the basis. Most of the students in the choice of physical education, there are blind class selection and other problems, only a few students because they like to choose badminton class. Most students choose Badminton Class with the same mentality because they don’t have special favorite sports. These students have no clear learning motivation, just to meet the school’s assessment credit requirements for physical education, just to cope with the exam.^[5]^ The proportion of female students in badminton class is very high. They think badminton class is much easier than other sports. Physical education in Colleges and universities is an open activity for all students. The purpose is to let students master suitable exercise methods and cultivate lifelong physical spirit by participating in physical education. Therefore, students’ interest in a sport is an important factor that cannot be ignored.

### 3.2. Despise process and value results

For many years, university teachers have been influenced by the traditional teaching concept, and have been adopting the indoctrination teaching method. Among them, badminton teaching is the same. Teachers usually use the teaching method of explaining first, then let students’ practice. In order to consider the progress of the course, the teacher takes up most of the time in the class and leaves little time for the students to practice. At the end of the term, teachers will grade students according to their own assessment standards. Under this teaching mode, students are particularly passive in accepting knowledge. Even if some students are very attentive in the whole learning process, they cannot get the attention of teachers. Many students have the problem that they cannot get good grades due to various reasons.^[6]^ Therefore, students’ enthusiasm for badminton lessons will gradually decrease, and their mentality will also be affected. In the end, the students just try to master some skills in order to cope with the final exam. This kind of learning method lost the original intention of choosing badminton class because of interest and hobbies, and the enthusiasm will gradually fade. The students’ psychology has changed from loving badminton to dislike, even to repel. As a result, badminton classroom atmosphere is poor, the interaction between students and teachers will also be reduced, reducing the teaching effect.

### 3.3. Belittle features and value content

At present, there are some problems in Badminton Class in Colleges and universities, which despise the characteristics of badminton and pay too much attention to the content. In order to make the students get high marks in the final exam, the teacher pays close attention to the practice of basic skills in the classroom. Most of the teachers still use the traditional single boring teaching methods to carry out indoctrination teaching. Students can only take part in the exam for the sake of coping with the exam. They do not like badminton from the heart. All these factors have seriously affected the development of students’ individualized sports. At the same time, when many colleges and universities evaluate badminton course, the content of the assessment is complex. It includes racket holding technique, serving technique, and hitting technique, as well as smashing and smashing technique, backhand net shooting technique and so on.^[7]^ For badminton, students’ speed and endurance quality is very important. At the same time, badminton requires high flexibility for learners, such as changing left and right hands and backcourt. In addition, badminton also needs good coordination, reaction speed, insight, and strength. However, there are very few contents about the assessment reflecting the characteristics of badminton, and the proportion of assessment score is very low. Therefore, the assessment score cannot reflect the characteristics of badminton class, which makes the significance of learning badminton class greatly reduced.

## 4. A new way to improve the evaluation standard of college badminton

### 4.1. The content of examination should be free to improve students’ interest in study

In badminton teaching, teachers should strengthen their guiding role, let students as the main body of the classroom, let them participate in it independently, to stimulate students’ love and passion for badminton. Teachers should guide students to participate in the classroom exercise, deepen the impression of badminton from their own experience process, and feel the joy brought by sports. Only by arousing students’ passion for badminton can they correct their learning motivation and attitude, and then better cultivate the lifelong sports spirit. At the same time, colleges, and universities, based on the actual situation and the characteristics and professionalism of badminton, set up more entertaining assessment content. The main characteristic of these examination contents is that the requirements for technology and physical quality of students are relatively low. In badminton assessable scoring, students can choose 2 or more items as the test content according to their own situation and mastery. In this way, students can not only avoid inferiority, but also feel the joy of harvest. Using such assessment and scoring standards can improve students’ sports level and stimulate students’ interest in badminton and other sports. Flexible assessment and scoring standards can guide, check, and urge the theoretical knowledge and practical methods of badminton class. To improve the teaching efficiency of physical education and cultivate the students’ lifelong physical spirit.

### 4.2. Pay attention to process evaluation and assessment

For college teaching, most of the teaching results of the course can not only be evaluated based on the final examination results. Especially for physical education, students’ daily performance is particularly important. Badminton learning is also a process sport. Therefore, when the badminton class is evaluated, it is unreasonable to only look at the result instead of the process. Badminton Class assessment score should increase the proportion of students’ daily performance score, which can help to cultivate students’ independent participation. Teachers should guide and help students to improve their self-evaluation ability, and then promote the development of physical exercise habits. Teachers can design some small-scale classroom teaching competitions to let students participate in them. Teachers and other students who are not involved in the evaluation can be graded according to the overall performance of the participants. Finally, the accumulated badminton course assessment score is regarded as an important reference for the final assessment score. Only in this way can we consider the objective reasons for the uneven physical quality of each student, so that the students with poor physical quality can also fully participate in the badminton class.

### 4.3. Establish a reasonable structure and proportion of performance evaluation

Badminton Class Evaluation and scoring standards can not only see the final examination results, but also pay attention to the level of progress of students. Teachers can rationalize the proportion of final exam scores, for example, the final exam score accounts for 60% of the total score, and the process evaluation is that the daily performance accounts for 40% of the total score. According to the actual situation, teachers can refine the process evaluation and take the advantages of each student into account. Especially for some students who are poor in physique, but work hard, process evaluation is very important. This can not only meet the development needs of different students, but also encourage the students with poor sports skills, to stimulate the enthusiasm of each student. Let students realize that if the learning attitude is correct, every effort can be rewarded, to achieve a certain sense of achievement. This can not only improve the comprehensive quality of students, but also help students to establish the correct values. In addition, new methods, such as human pose estimation, can be adopted to assess badminton course in colleges and universities to establish scientific and reasonable scoring standard.

## 5. Conclusion

Badminton course in colleges and universities is a favorite sport of students, but there are many unscientific places in the current assessment and scoring standards. Teachers pay too much attention to skills, results, and content in teaching, so that students take badminton class just to cope with the final exam, not to participate in sports and strengthen their health. Because there are many problems in the process of teaching and assessment and scoring system, students’ enthusiasm for badminton class is not high, and the teaching effect is poor. Therefore, colleges and universities should focus on innovation and improvement of the badminton course assessment and scoring standards, carry out the free reform of the assessment content, pay attention to the learning process of students, and formulate a reasonable evaluation structure, so as to stimulate students’ enthusiasm for learning, and then cultivate students’ lifelong sports spirit. The experimental methods of badminton course, such as randomized controlled trial, were not evaluated in our present study. Thus, additional work needs to be done to address this limitation in the future.

## Authors contributions

**Conceptualization:** Xiaofeng Hu.

**Data curation:** Xiaofeng Hu.

**Formal analysis:** Xiaofeng Hu.

**Investigation:** Xiaofeng Hu.

**Methodology:** Xiaofeng Hu.

**Writing – original draft:** Qiaolin Jiang.

**Writing – review & editing:** Xiaofeng Hu.
